# Antimicrobial Combined Action of Graphene Oxide and Light Emitting Diodes for Chronic Wound Management

**DOI:** 10.3390/ijms23136942

**Published:** 2022-06-22

**Authors:** Silvia Di Lodovico, Firas Diban, Paola Di Fermo, Morena Petrini, Antonella Fontana, Mara Di Giulio, Adriano Piattelli, Simonetta D’Ercole, Luigina Cellini

**Affiliations:** 1 Department of Pharmacy, University of “G. d’Annunzio” Chieti-Pescara, 66100 Chieti, Italy; silvia.dilodovico@unich.it (S.D.L.); firas.diban@unich.it (F.D.); paola.difermo@unich.it (P.D.F.); antonella.fontana@unich.it (A.F.); mara.digiulio@unich.it (M.D.G.); l.cellini@unich.it (L.C.); 2 Department of Medical, Oral and Biotechnological Sciences, University of “G. d’Annunzio” Chieti-Pescara, 66100 Chieti, Italy; morena.petrini@unich.it; 3 School of Dentistry, Saint Camillus International University of Health and Medical Sciences, Via di Sant′Alessandro 8, 00131 Rome, Italy; apiatteli51@gmail.com; 4 Faculty of Dental Medicine, University of Belgrade, 11000 Belgrade, Serbia; 5 Fondazione Villa Serena per la Ricerca, 65013 Città Sant′Angelo, Italy; 6 Casa di Cura Villa Serena del Dott. L. Petruzzi, 65013 Città Sant′Angelo, Italy

**Keywords:** chronic wounds, *Staphylococcus aureus*, *Pseudomonas aeruginosa*, graphene oxide, light emitting diodes, Lubbock chronic wound biofilm model, polymicrobial biofilm, antimicrobial resistance

## Abstract

Innovative non-antibiotic compounds such as graphene oxide (GO) and light-emitting diodes (LEDs) may represent a valid strategy for managing chronic wound infections related to resistant pathogens. This study aimed to evaluate 630 nm LED and 880 nm LED ability to enhance the GO antimicrobial activity against *Staphylococcus aureus-* and *Pseudomonas aeruginosa*-resistant strains in a dual-species biofilm in the Lubbock chronic wound biofilm (LCWB) model. The effect of a 630 nm LED, alone or plus 5-aminolevulinic acid (ALAD)-mediated photodynamic therapy (PDT) (ALAD-PDT), or an 880 nm LED on the GO (50 mg/l) action was evaluated by determining the CFU/mg reductions, live/dead analysis, scanning electron microscope observation, and reactive oxygen species assay. Among the LCWBs, the best effect was obtained with GO irradiated with ALAD-PDT, with percentages of CFU/mg reduction up to 78.96% ± 0.21 and 95.17% ± 2.56 for *S. aureus* and *P. aeruginosa*, respectively. The microscope images showed a reduction in the cell number and viability when treated with GO + ALAD-PDT. In addition, increased ROS production was detected. No differences were recorded when GO was irradiated with an 880 nm LED versus GO alone. The obtained results suggest that treatment with GO irradiated with ALAD-PDT represents a valid, sustainable strategy to counteract the polymicrobial colonization of chronic wounds.

## 1. Introduction

Chronic wounds are defined as those that do not heal in an orderly and timely way within three months, representing an important challenge worldwide. They are characterized by excessive levels of pro-inflammatory cytokines, proteases, reactive oxygen species (ROS), and senescent cells, as well as the existence of persistent infections [1].

In a chronic wound, polymicrobial biofilms play a pivotal role in the pathogenesis of wounds, impairing cutaneous healing [2]. In particular, in the polymicrobial sessile growth mode, different bacterial species communicate, cooperate, or compete with each other. Wounds’ microbial colonization involves different aerobic and anaerobic pathogenic microorganisms including bacteria and yeasts. *Staphylococcus aureus* and *Pseudomonas aeruginosa* are the main pathogens isolated in chronic polymicrobial infections [3]. In an early phase of colonization, an antagonistic interaction occurs and *P. aeruginosa* tries to kill *S. aureus*; then, a synergistic interaction occurs with an increasing tolerance of traditional treatments [4]. Polymicrobial infections are more persistent than mono-microbial ones. In fact, Orazi et al. [5] reported that the *P. aeruginosa* exoproducts decrease the susceptibility of *S. aureus* to vancomycin and tobramycin due to *P. aeruginosa* 4-hydroxy-2-heptylquinoline-N-oxide (HQNO) production. Moreover, the release of N-acetyl glucosamine (GlcNAc) by *S. aureus* stimulates the *P. aeruginosa* quinolone signal (PQS), which controls the extracellular virulence factors’ production (e.g., pyocyanin, elastase, rhamnolipids, and HQNO) and quorum sensing [5,6]. In a typical chronic wound, *P. aeruginosa* colonizes the deep wound and *S. aureus* is found at the surface. *Pseudomonas aeruginosa* can colonize the deeper region of the chronic wound thanks to its capability to migrate via type-IV pili and flagellum-mediated motility in biofilms [7].

A suitable in vitro model to study the effect of novel solutions to counteract chronic wound infections is the Lubbock chronic wound biofilm (LCWB) model. Since the media used contains red blood cells, plasma, and nutrients in a typical 3D gradient, this model mimics the microbial distribution and environment of a real chronic wound [8,9,10]. In particular, *S. aureus* produces a fibrin network that represents a scaffold on which bacteria can adhere, forming a biofilm. The LCWB is a well-recognized model for evaluating the interactions among microorganisms from a chronic wound, including aerobic and anaerobic microorganisms. Consequently, before conducting in vivo studies, this complex model is used to evaluate the effects of different antimicrobials on microorganisms in a complex chronic wound-resembling environment [11].

Different treatments are proposed to prevent the bacterial infection of skin wounds, such as iodine, silver, zinc oxide, and polyhexamethylene, but they have certain levels of cytotoxicity [6]. Moreover, the increasing number of multidrug-resistant strains strongly suggests the need for new and more effective antimicrobials for clinical application. We previously demonstrated the effect of graphene oxide (GO) in an LCWB model against clinical *S. aureus* and *P. aeruginosa* strains. Graphene oxide reduces the growth of *S. aureus* and *P. aeruginosa* by wrapping up the microorganisms and increasing the fluidity of the fibrin network in LCWBs [3]. Moreover, GO has been recognized as a smart and cheap material with wide application potential [12]. In the literature, it has been reported that GO is activated by light-emitting diodes (LEDs) [13]. In this regard, the LEDs, semiconductors that convert electrical current into incoherent narrow-spectrum light, represent a new treatment against microorganisms that are difficult to treat. In fact, LEDs show good antimicrobial action against Gram-positive and -negative bacteria, alone or combined with chemical antimicrobials such as sodium hypochlorite and chlorhexidine [14]. Light-emitting diodes are widely used in clinical therapy for pain attenuation, wound healing, skin rejuvenation, and repair [15]. Light-emitting diodes, emitting red light with wavelengths between 610 and 760 nm, are made up of semiconductors of different materials: aluminum gallium arsenide, gallium arsenide phosphide (GaAsP), aluminum gallium indium phosphide (AlGaInP), gallium phosphide (GaP), or aluminum gallium arsenide (AlGaAs). In terms of clinical applications, red LEDs are widely used in dentistry, algaculture, and wound healing [16]. Infrared light (>760 nm) LEDs are used for home-entertainment remotes, night-vision cameras, security systems and medical devices for promoting tissue regeneration and healing. Nowadays, the use of photodynamic therapy represents an eco-sustainable and efficacious therapy to counteract the worrying phenomenon of infections related to resistant microorganisms [17]. Yang et al. [18] demonstrated the efficacy of the synergistic combination of the photodynamic/photothermal properties of diketopyrrolopyrrole and fluconazole against resistant *Candida albicans* infections. Moreover, LEDs are characterized by an antibacterial activity given they can excite endogenous photosensitive compounds, e.g., porphyrins, present in the bacterial cells, causing the production of ROS such as hydroxyl radicals, hydrogen peroxide and singlet oxygen (^1^O_2_). Reactive oxygen species further react with cellular components, causing cell death [19]. Aminolevulinic acid (ala), a precursor of the natural photosensitizer protoporphyrin IX (PpIX), can be administered exogenously to reduce the accumulation of the molecules and cytotoxic effects of irradiation. An increase in photoactive porphyrin products (PAPs) produces a bacteria-killing action [20]. 

Based on these considerations, this work aimed to study the antimicrobial combined effect of GO and 630 nm LEDs, alone or with ala (ALAD-PDT), and at 880 nm in the double-species *S. aureus* and *P. aeruginosa* LCWB model.

**Figure 1:**
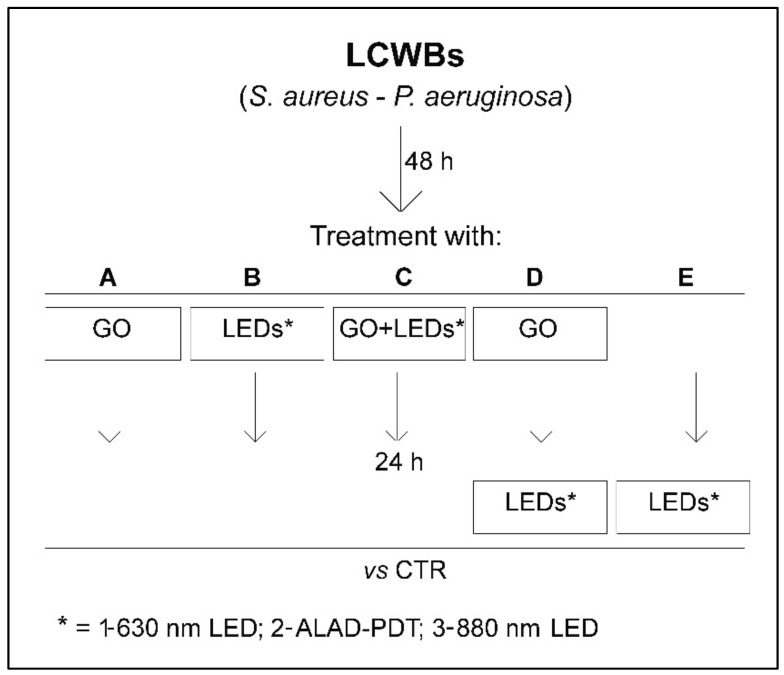
Experimental plan of the study. The mature LCWBs, obtained after 48 h of incubation, were placed on a “wound bed” and treated as follows: A = with GO (50 mg/mL) and incubated for 24 h; B1 = with 630 nm LED for 17 min and incubated for 24 h; B2 = with ALAD-PDT for 17 min and incubated for 24 h; B3 = with 880 nm LED for 17 min and incubated for 24 h; C1 = with GO (50 mg/mL) irradiated with 630 nm LED for 17 min and incubated for 24 h; C2 = with GO (50 mg/mL) irradiated with ALAD-PDT for 17 min and incubated for 24 h; C3 = with GO (50 mg/mL) irradiated with 880 nm LED for 17 min and incubated for 24 h; D1 = with GO (50 mg/mL) incubated for 24 h and then irradiated with 630 nm LED for 17 min; D2 = with GO (50 mg/mL) incubated for 24 h and then irradiated with ALAD-PDT for 17 min; D3 = with GO (50 mg/mL) incubated for 24 h and then irradiated with 880 nm LED for 17 min; E1 = with 630 nm LED for 17 min after 24 h of incubation; E2 = with ALAD-PDT for 17 min after 24 h of incubation; E3 = with 880 nm LED for 17 min after 24 h of incubation. The untreated mature LCWBs (CTRs), after 48 h of incubation, were placed on a “wound bed” and incubated for 24 h in the same time and temperature conditions as all experimental LCWBs.

The experimental plan was organized as follows (Figure 1).

## 2. Results

The combined effect of GO and LEDs was evaluated on *S. aureus* and *P. aeruginosa* growth in the LCWB model. The resistant *S. aureus* and *P. aeruginosa* clinical strains used for the experiments were characterized for their main virulence factors (Supplementary Table S1).

**Figure 2:**
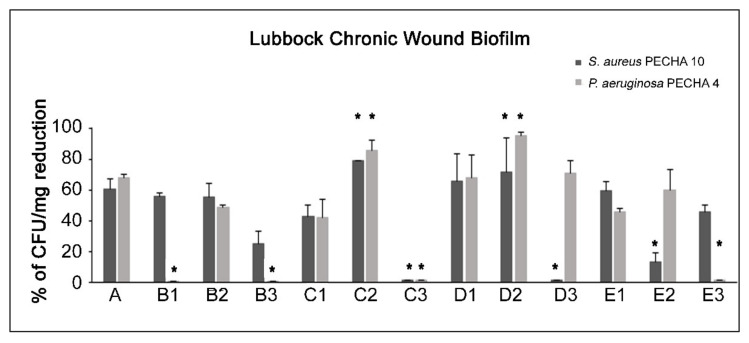
Percentages of CFU/mg reduction of *S. aureus* and *P. aeruginosa* in treated LCWB model versus the control. For the experimental points, see Figure 1 and the Section 4, paragraph 5. * Statistically significant difference (*p* < 0.05) for each strain in respect to A condition.

Figure 2 shows the percentages of CFU/mg reduction of *S. aureus* and *P. aeruginosa* in the LCWB model after treatment with GO and LEDs. 

In the B1 condition, a remarkable *S. aureus* CFU/mg reduction versus the control (*p* < 0.05) was generated without affecting *P. aeruginosa.* A CFU/mg reduction was produced for both tested strains in the B2 condition, meanwhile, with a significant effect versus the control (*p* < 0.05) but not versus GO. When the LCWB was treated in the B3 condition, weak *S. aureus* growth reduction was noted. 

As shown in Figure 2, in the C1 combination, an antimicrobial on the growth of *S. aureus* and *P. aeruginosa* was observed in the LCWB model without a statistically significant (*p* > 0.05) effect versus GO. When the LCWB was treated under the C2 combination, significant (*p* < 0.05) CFU/mg reductions for *S. aureus* (78.96% ± 0.21) and *P. aeruginosa* (85.67% ± 6.56) were detected versus the control and the A condition. On the contrary, the LCWB treated under the C3 combination showed an antagonist effect, without CFU/mg reductions for any tested strain. 

The LCWB treated with the D1 combination showed greater antimicrobial efficacy than the C1 combination and a similar trend to the A condition. The treatment with the D2 combination increased the *P. aeruginosa* growth effect with significant (*p* < 0.05) CFU/mg reduction (95.17% ± 2.66) versus the control and the A condition. For *S. aureus*, a slightly greater CFU/mg reduction was produced versus the other combinations, with a statistically significant difference (*p* < 0.05) versus the control.

The treatment with the D3 combination displayed an effect only against *P. aeruginosa* growth, with significant differences (*p* < 0.05) when compared to the control, B3 condition, and C3 combination.

A similar effect to the A condition was obtained under the E1 condition, with 60.10% ± 5.81 and 45.82% ± 1.81 reductions in *S. aureus* and *P. aeruginosa*, respectively. In E2, a similar trend to the A condition was recorded only for *P. aeruginosa*. A good *S. aureus* CFU/mg reduction was detected in the E3 condition with no significant difference compared to the A condition.

Considering all tested conditions, the best enhancement of GO antimicrobial action was obtained via irradiation with ALAD-PDT (C2 and D2 combinations).

**Figure 3:**
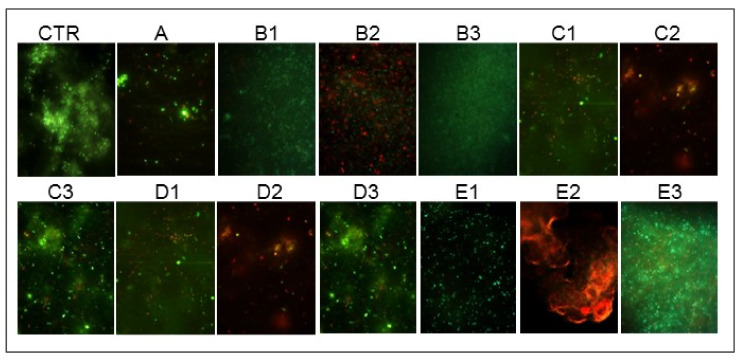
Representative live/dead images of untreated (CTR) and treated LCWBs. For the experimental points, see Figure 1 and the Section 4, paragraph 5.

No reduction in the cell number and viability versus the control were detected with 630 nm and 880 nm LEDs in the B1 and B3 conditions. A remarkable number of red cells was recorded in the B2 condition with the ALAD application. In general, the cell viability in the presence of GO was influenced by the LEDs. In particular, in the C1 and C2 combinations, the cell clusters appeared more disaggregated and there were more dead cells compared to the control and the A condition. In the presence of ALAD (C2, D2, E2), the cells were blocked by the gel and most were dead (red). The images obtained with the C3 and D3 combinations confirmed the CFU/mg results, with more green cells versus the A condition and C1 and C2 combinations. In the D1 and D2 conditions, the number of damaged cells increased and their number was fewer than in both the control and other conditions. In E1 and E3, the treatment with LEDs did not affect the cell viability (Figure 3).

**Figure 4:**
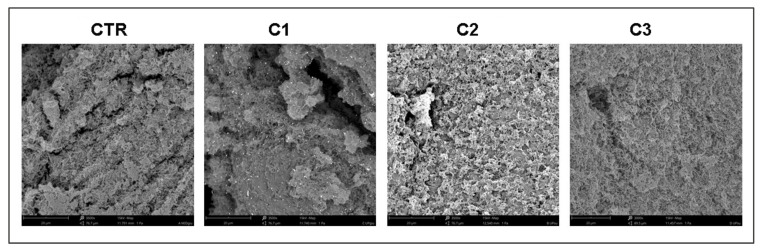
Representative SEM images of untreated (CTR) and treated LCWBs treated with C1, C2, and C3 combinations. For the experimental points, see Figure 1 and the Section 4, paragraph 5.

The representative SEM images (Figure 4) showed non-mixed bacteria in the untreated LCWB (CTR) with a prevalence of bacillary *P. aeruginosa* cells. In the C1 combination, a weak bacterial reduction was detected with cleavage plans of the biomass biofilm versus the control. A remarkable strain reduction was displayed in the LCWB treated with the C2 combination, confirming a significantly greater antimicrobial potentiating effect of 630 nm LEDs toward GO. No difference in terms of microbial reduction was noted for the LCWB treated with C3.

**Figure 5:**
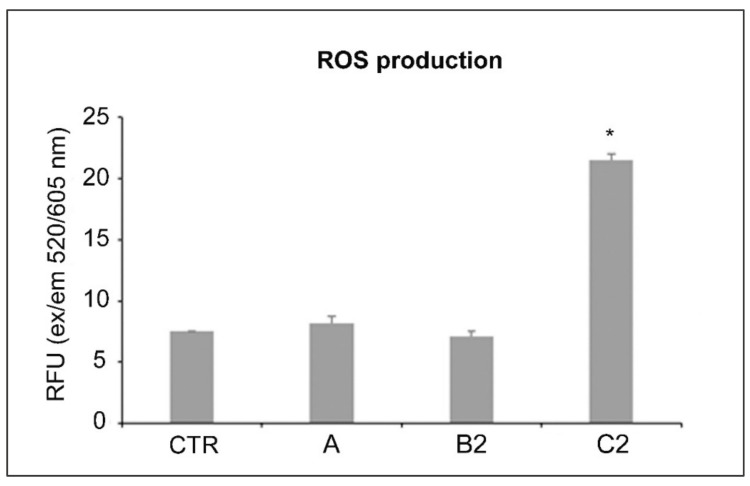
ROS production for the LCWBs: untreated (CTR), treated with the A condition, and treated with the B2 and C2 combinations. For the experimental points, see Figure 1 and the Section 4, paragraph 5. * Statistically significant difference from the CTR (*p* < 0.05).

The ROS production was normalized based on the weight of the LCWBs. As can be seen in Figure 5, the ROS evaluation showed significant (*p* < 0.05) ROS production in presence of GO plus ALAD-PDT, confirming the strong antimicrobial action of this combination. No statistical significance (*p* > 0.05) was obtained with GO and 630 nm LEDs alone, either in respect to the control or each other.

## 3. Discussion

Uncovering new non-antibiotic strategies to counteract microbial proliferation in chronic wounds was the pivotal aim of this study. The use of antibiotics for chronic wound management is not ideal and the search for new non-antibiotic strategies has been well considered by the WHO [21]. The failure of standard therapies can be associated with an increase in the resistant/tolerant microorganisms in polymicrobial biofilms. The co-infection of multiple strains in the biofilm environment leads to suppression of the immune response and wound healing activities, contributing to enhancements of both the antibiotic resistance/tolerance and virulence factors’ production. Hence, it is essential to identify novel solutions to overcome this issue [22]. 

In the present study, we evaluated the antimicrobial/antibiofilm/antivirulence action of GO on the polymicrobial LCWB model when irradiated with LEDs in different experimental conditions. Graphene oxide’s unique structure gives it several important properties. Graphene oxide is a one-atom-thick carbon-based nanomaterial with several oxygen-containing groups, a large surface area, and high near-infrared absorbance [17]. Generally, LED therapy relies on photosensitizer material being excited by irradiation, followed by an interaction with oxygen-containing functionalities in situ, causing cell death with different mechanisms [23]. Several applications of this interesting technology have been studied thoroughly, starting with tumor therapy and targeting, immune system response modulation, tissue regeneration, periodontal diseases, and wound infections [24,25,26]. In particular, in the present work, enhanced GO action was obtained when it was combined with ALAD-PDT. The microbial CFU/mg reduction in the presence of GO plus ALAD-PDT could be justified by the expected activation of GO through irradiation, leading to stimulated ROS production (especially oxygen singlet ^1^O_2_) [13]. This hypothesis is demonstrated by an increase in ROS production versus the control and versus GO and 630 nm LED alone. The same results obtained with GO plus ALAD-PDT at the two exposure times show a double application of the LEDs: an immediate enhancement of the antimicrobial GO effect and preservation of the GO effect over time. As such, if immediate antimicrobial action is required, the 630 nm LED can be irradiated immediately after GO treatment; if preservation of the GO effect over time is required, after the treatment with GO, it is possible to apply the 630 nm LED. This major killing effect compared to the other setups is due to the activation of both GO and ALAD by irradiation. Greater activation products were available in the biofilm model, creating a stressful environment that promotes cells’ death. Aladent, as a photosensitizer, acts as an exogenous prodrug that inters the bacterial cells, causing accumulation in 5-aminolevulinic acid, which overwhelms the enzymatic capacity in the heme synthesis pathway, resulting in protoporphyrin IX (PpIX) accumulation. Finally, PpIX, under irradiation, serves as an in situ photosensitizer, leading to bacterial inactivation [27]. In an in Vitro study, Petrini et al. [28] demonstrated the increase in fluorescence of PpIX after the addition of ALAD gel to *E. faecalis* solution and after the LED irradiation, the endogenous PpIX produced remarkable cell death.

Several studies mentioned the LED therapy impact in different conditions against biofilm-forming microorganisms associated with chronic wound infections.

Elias et al. [29] indicated the remarkable effect of LED therapy (up to 3 h of application) after GO treatment against methicillin-resistant *S. aureus* without using a photosensitizer. Mahmoudi et al. [30] found an important reduction in the expression of biofilm-associated *S. aureus* genes after applying 630 nm LED irradiation with a photosensitizer (toluidine blue O).

Similar results were obtained in Tan et al.’s study [31], in which ALAD-PDT application caused the destruction of the biofilm structure and reduced the expression of quorum-sensing genes in *P. aeruginosa*. Meanwhile, our study outcomes provided interesting results about this effect in the LCWB model, confirming that irradiation acted on the complex biofilm, taking into consideration the mutual interaction between bacteria in the biofilm and the bacterial distribution in the artificial wound model.

In presence of infrared LEDs and GO, there was an antagonistic action in a dual-species polymicrobial biofilm compared to the treatment with 880 nm LED and GO alone. This finding could seem to contradict those of Petrini et al. [32], but we must consider that the LCWB is a complex 3D polymicrobial biofilm in which the synergistic interaction of *S. aureus* and *P. aeruginosa* increases tolerance. LED treatment can produce oxidative stress, protein damage, and inhibition or delay of growth, without killing the microorganisms that were irradiated, which remained viable (green) [33]. The microbial population decreased, indicating a growth inhibition effect versus the control, without a killing action. Instead, remarkable cell death was detected in presence of ala. As demonstrated by Petrini et al. [28], the gel could englobe and suffocate the cells most susceptible to the treatment.

Through SEM analysis, in the samples treated with GO plus ALAD-PDT, more areas free of bacteria were observed than in the control. These SEM results were in agreement with Li et al., who demonstrated the effect of ALAD-PDT in reducing adherent and aggregated bacteria, suggesting that ALAD-PDT could cause cell detachment, and consequently, inhibition of biofilm formation [34].

The interesting antibacterial results obtained with GO activated by LEDs are of particular importance considering the action of LEDs on regeneration. In fact, as demonstrated by Yang et al. [35], irradiation with ALAD-PDT significantly increased the re-epithelialization of the wound in mice, with a reduction of the NF-κB signaling pathway involved in acute and chronic inflammatory responses.

Future studies should be performed on epithelial cells to confirm these results, including GO.

In conclusion, GO treatment with ALAD-PDT irradiation could represent a valid strategy to counteract the microbial proliferation of chronic wound microorganisms. Furthermore, this therapy represents a valid ecofriendly strategy with a low environmental impact in terms of the manufacturing, transport, and disposal for each production cycle. In terms of application, for the treatment of microbial proliferation in the LCWB, we suggest applying GO irradiated immediately with 630 nm LED and reprocessed with 630 nm LED after another 24 h, to increase the effectiveness of the GO. The results suggest the creation of advanced medicaments consisting of GO and 630 nm LED or only with GO and then treated with 630 nm LED devices. Noteworthy is the low cost of both GO and the 630 nm LED, giving this research a great economic and public health impact.

## 4. Materials and Methods

### 4.1. Bacterial Cultures

Anonymized clinical strains *S. aureus* PECHA 10 and *P. aeruginosa* PECHA 4, isolated from patients with chronic wounds, were used in this study (Inter-Institutional Ethic Committee of University “G. d’Annunzio” Chieti-Pescara, Chieti, Italy, ID n. richycnvw). These bacteria, coming from the private collection of the Bacteriological Laboratory of the Pharmacy Department, University “G. d’Annunzio” Chieti-Pescara, were cultured on Mannitol Salt Agar (MSA, Oxoid, Milan, Italy) and Cetrimide Agar (CET, Oxoid, Milan, Italy), respectively. The bacteria were previously characterized for their resistance profiles [36]. In this study, *S. aureus* PECHA 10 was characterized for its hemolytic activity, detection of *agr* allels and *icaA/icaD* genes, and biofilm production [37]. Briefly, for the hemolytic activity, 5 µL of refreshed *S. aureus* PECHA 10 broth cultures in Tryticase Soy Broth (TSB, Oxoid, Milan, Italy) (OD_600_ = 0.04–0.05) were spotted onto Trypticase Soy Agar (TSA, Oxoid, Milan, Italy) plus 5% sheep sterile blood (Biomerieux, Florence, Italy) and incubated for 48 h at 37 °C, then chilled at 4 °C for 1 h for the evaluation. For the *agr* genotyping, a multiplex polymerase chain reaction (PCR) was performed according to Di Stefano et al. [38], and the presence of *icaA* and *icaD* was detected by PCR according to Zhou et al. [39]. *Pseudomonas aeruginosa* was characterized for the *lasB* gene according to Lanotte et al. [40], and for its capability to form a biofilm.

For the study, the bacteria were cultured in TSB and incubated at 37 °C overnight in an aerobic condition, and then refreshed for 2 h at 37 °C in an orbital shaker in an aerobic condition. The cultures were standardized to an optical density at 600 nm (OD_600_) = 0.125 and diluted 1:10 for *S. aureus* PECHA 10 and 1:100 for *P. aeruginosa* PECHA 4, to obtain 10^6^ and 10^5^ CFU/mL, respectively [3].

### 4.2. Substances

#### 4.2.1. Preparation of Graphene Oxide Aqueous Dispersion

Graphene oxide (GO) in an aqueous solution of 4 g/L (Graphenea, Donostia San Sebastian, Spain) was added to PBS (Merk, KGaA, Darmstadt, Germany) to obtain the desired concentration, bath ultrasonicated for 10 min (37 kHz, 180 W; Elmasonic P60H; Elma), and sterilized for 2 h under a UV lamp (6 W, 50 Hz, 0.17 A; Spectroline EF 160/C FE; Spectronics). The GO concentration was standardized spectrophotometrically at λmax 230 nm. Graphene oxide flakes’ dimensions (dimension: 670 ± 50 nm at 37 °C; polydispersity: 0.25 ± 0.02) were checked using dynamic laser light scattering (DLS) (90Plus/BI-MAS ZetaPlus multi-angle particle size analyzer; Brookhaven Instruments Corp.) [3]. For the study, GO was used at the non-toxic concentration of 50 mg/L [41].

#### 4.2.2. Aladent Gel

Aladent (ALAD) (Alpha strumenti, Melzo, Milan, Italy) is a gel containing 5% of 5-aminolevulinic acid (ala), as previously described. ALAD is covered by a patent (PCT/IB2018/060368, 12.19.2018) where the object is a “pharmaceutical preparation comprising a topically released active ingredient and a heat-sensitive carrier, method of obtaining same, and use of same in the treatment of skin and mucosal infections”. The full text is registered on patentscope/WIPO.int with pub. no. WO2019123332 [20,28,42].

### 4.3. Light-Emitting Diode (LED) Devices

In this experiment, two different devices emitting light at different wavelengths were applied for 17 min, a time included in the previously evaluated time ranges (from 5 to 20 min) and recommended by the manufacturer (TR-LUX, Errevi group, Bergamo, Italy). The following instruments were used:

- An AlGaAs power LED device (TL-01), characterized by a 630 nm ± 10 nm FHWM nm wavelength, was used as light source (Alpha strumenti, Melzo, Milan, Italy). The handpiece constituted by one LED with a 6-mm diameter at the exit and a surface irradiance of 380 mW/cm^2^. During the experiments, the LED handpiece was mounted perpendicularly to the LCWB sample at a 0.5-mm distance. Irradiation was performed under a laminar flow hood in the dark, under aseptic conditions [20,28,42]. For the tests, 630 nm LED alone and plus ALAD (ALAD-PDT) were used.
- A NIR-LED device characterized by an 880 nm wavelength was used as the light source TR-LUX (Errevi, Bergamo, Italy). The handpiece constituted six LEDs (12 mm diameter) disposed in two lines. Each of the by six LEDs was used for irradiation, emitting a power output of 2.37 mW [14,25].

For both devices, light irradiation was performed by keeping the light sources stationary, in a perpendicular position, and at 0.5 mm from the samples.

### 4.4. Lubbock Chronic Wound Biofilm (LCWB) Model

The LCWB model was prepared according to Di Giulio et al. (2020) [3]. In brief, 5 mL of medium containing Brucella Broth (BB, Oxoid, Milan, Italy) with 0.1% agar bacteriological, 50% porcine plasma (Sigma Aldrich, Milan, Italy), 5% horse erythrocytes (BBL, Microbiology System, Milan, Italy) and 2% fetal calf serum (Biolife Italiana, Milan, Italy) were distributed in sterile glass tubes. For the LCWB preparation, 10 µL of each diluted broth culture were inoculated in glass tubes with sterile pipette tips. After 48 h of incubation, the mature biofilm was harvested from the glass tubes, the pipette tip was removed, the biofilm biomass was washed two times with sterile PBS, and the LCWB volumes were determined [3]. The LCWBs were placed on a “wound bed”, an artificial home-made oval-shaped wound bed created with sterile 1.5 mL Eppendorf tubes on the surface of a nutrient medium containing Bolton Broth (Oxoid, Milan, Italy) with 1.5% agar, to reproduce an in Vitro chronic wound biofilm and to allow for the treatment [3]. 

### 4.5. Effect of GO Alone and Combined with LEDs on LCWB Model

The effect of GO alone and combined with LEDs was evaluated following the experimental plan displayed in Figure 1. In detail, the mature LCWBs, incubated for 48 h at 37 °C in an aerobic condition, were differentiated as follows:

**A** = treated with:

GO (50 mg/L) and incubated for 24 h at 37 °C in an aerobic condition and then analyzed vs. the control;

**B** = irradiated for 17 min with:

- B1: 630 nm LED
- B2: ALAD-PDT
- B3: 880 nm LED

and incubated for 24 h at 37 °C in an aerobic condition and then analyzed vs. the control;

**C** = treated with:

GO (50 mg/L) and irradiated for 17 min with:

- C1: 630 nm LED
- C2: ALAD-PDT
- C3: 880 nm LED

then incubated for 24 h at 37 °C in an aerobic condition and then analyzed vs. the control;

**D** = treated with:

GO (50 mg/L), incubated for 24 h at 37 °C in an aerobic condition and then irradiated for 17 min with:

- D1: 630 nm LED
- D2: ALAD-PDT
- D3: 880 nm LED

then analyzed vs. the control;

**E** = incubated for 24 h at 37 °C in an aerobic condition and then irradiated for 17 min with:

- E1: 630 nm LED
- E2: ALAD-PDT
- E3: 880 nm LED

then analyzed vs. the control.

All experimental points (A–E) were analyzed after 72 h (48 h for the mature LCWB preparation plus 24 h for the treatments).

The effect of GO alone and combined with LEDs was evaluated in terms of: (i) *S. aureus* PECHA 10 and *P. aeruginosa* PECHA 4 CFU/mg reduction; (ii) live/dead analysis; (iii) scanning electron microscope (SEM) evaluation; iv) ROS production.

For CFU/mg determination, after treatment and incubation, the biofilm was harvested from the artificial “wound bed”, washed twice with sterile PBS, and the weight was measured. Subsequently, the biofilm was vortexed for 2 min, sonicated for 3 min (with ultrasound bath), vortexed for other 2 min and diluted in PBS for the microbial enumeration. Live/dead staining was used to confirm the effect of this procedure in terms of disaggregating action and cell viability retention. The CFU/mL was determined by spreading on MSA for *S. aureus* PECHA 10 and on CET for *P. aeruginosa* PECHA 4 and the plates were incubated at 37 °C for 24–48 h. Data were expressed as percentage of CFU/mg reduction versus the control.

For the cell viability, observation under fluorescence Leica 4000 DM microscopy after live/dead staining was performed according to Di Giulio et al. (2020) [3].

The SEM observation was carried out according to Di Fermo et al. [43]. Briefly, the untreated and treated LCWBs were fixed with glutaraldehyde, dehydrated with ascending concentrations of ethanol and then immersed in hexamethyldisilazane (HMDS, Sigma-Aldrich, Milan, Italy) for 10 min, twice. Hexamethyldisilazane was decanted from the specimen vial and the tissues were left to air dry at room temperature. The dried samples were subjected to the gold-sputtering with a Desk Sputter Coater (Phenom-World B.V., Dillenburgstraat 97 Eindhoven, 5652,AM, Netherlands) and then observed under SEM (Phenom-World B.V., Dillenburgstraat 97 Eindhoven, 5652,AM, Netherlands) at different magnifications.

The best-performing combinations were analyzed for ROS production to define a possible mechanism of action. The ROS production in cells was detected using a Cellular Reactive Oxygen Species Detection Assay Kit (ab186027, Abcam, Cambridge, UK), following the manufacturer’s instructions. Briefly, after treating LCWBs with the best-performing GO and LED combinations, the cells were seeded into a 96-well plate × cells per well. We added 100 µL/well of ROS Red Working Solution to the plate. Then, we incubated the cell plate at 37 °C for 1 h and we detected the fluorescence signal using a microplate reader (Synergy H1 BioTek, Santa Clara, CA 95051, USA) after the treatments. 

### 4.6. Statistical Analysis

Data were obtained from at least three independent experiments performed in duplicate. Data were shown as the means ± standard deviation. Differences between groups were assessed with one-way analysis of variance (ANOVA). *P*-values ≤0.05 were considered statistically significant.

## 5. Conclusions

The present study underlines the notable action of non-antibiotic compounds against resistant pathogens mainly growing in polymicrobial biofilms, involved in chronic wound infections. The combined use of GO plus ALAD-PDT represents a valid suggestion for chronic wound management. An added value of these promising results relates to the eco-sustainability of the proposed non-antibiotic combinations: the evolutionary smart low-cost of GO and the low environmental impact of the LED technology.

## 6. Patents

ALAD is covered by a patent (PCT/IB2018/060368, 12.19.2018) where the object is a “pharmaceutical preparation comprising a topically released active ingredient and a heat-sensitive carrier, method of obtaining same, and use of same in the treatment of skin and mucosal infections”. The full text is registered on patentscope/WIPO.int with pub. no. WO2019123332.

## Data Availability

Not applicable.

## Supplementary Materials

The following supporting information can be downloaded at: www.mdpi.com/article/10.3390/ijms23136942/s1. (Reference [37] is cited in the Supplementary Materials).
