# Supplementary material for: Antimicrobial Combined Action of Graphene Oxide and Light Emitting Diodes for Chronic Wound Management
Source: Int J Mol Sci. 2022 Jun 22;23(13):6942. doi: 10.3390/ijms23136942 (PMC9266944; doi:10.3390/ijms23136942)
Supplement: Supplementary file 1 [file ijms-23-06942-s001.zip › ijms-1776053-supplementary.pdf]

International Journal of
Molecular Sciences

Supplementary Material

Table S1. *Staphylococcus aureus* PECHA 10 and *Pseudomonas aeruginosa* PECHA 4 characterization for their capability to form biofilm and their main virulence factors.

Strains	Biofilm Producer *	Gene Detection	Hemolytic Action
S. aureus PECHA 10	+++	*agr1*; *icaA*/*icaD*	-
P. aeruginosa PECHA 4	+++	*lasB*	ND

ND= not detected.

* according to Stepanovic et al. [37].

Reference

37 Stepanović, S.; Vuković, D.; Dakić, I.; Savić, B.; Švabić-Vlahović, M. A Modified Microtiter-Plate Test for Quantification of Staphylococcal Biofilm Formation. *J. Microbiol. Methods* **2000**, 40 (2), 175–179. https://doi.org/10.1016/s0167-7012(00)00122-6.
